# Air quality in a revitalized special economic zone at the center of an urban monocentric agglomeration

**DOI:** 10.1038/s41598-024-66255-y

**Published:** 2024-07-05

**Authors:** Robert Cichowicz, Maciej Dobrzański

**Affiliations:** Faculty of Civil Engineering, Architecture and Environmental Engineering, Lodz University of Technology, Al. Politechniki 6, 90-924 Lodz, Poland

**Keywords:** PM_10_, PM_2.5_, SO_2_, H_2_S, Outdoor air quality, Urban planning, City revitalization, Environmental impact, Chemical engineering

## Abstract

In this study, we have examined the air quality within a revitalized, post-industrial urban area in Łódź, Poland. The use of Dron technology with mobile measurement equipment allowed for accurate assessment of air quality (particulate matter and gaseous pollutants) and factors influencing air quality (wind speed and direction) on a local scale in an area of 0.18 km^2^ and altitudes from 2 to 50 m. The results show that the revitalization carried out in the Lodz special economic zone area contributed to eliminate internal air pollution emitters through the use of ecological and effective heat sources. The exceedances permissible concentration values were local, and concerned mainly the higher measurement zones of the troposphere (more than 30 m above ground level). In the case of gaseous pollutants, higher wind speeds were associated with a decrease in the concentration of SO_2_ and an increase in H_2_S concentration. In both cases, the wind contributed to the occurrence of local areas of accumulation of these gaseous pollutants in the spaces between buildings or wooded areas.

## Introduction

Cities are constantly subject to economic, spatial, and functional transformations^1,2^. These changes particularly affect post-industrial cities, which, as a result of limiting or liquidating industrial activities, have lost their economic base. In response, there has been increasing emphasis on the renovation of existing urban resources, the renewal of city centers, and improving the quality of public spaces (revitalization)^3,4^. One example is the city of Łódź in central-eastern Poland. Since the geopolitical transition in 1991, Łódź has experienced significant demographic changes, with its population declining from 850,000 in 1988 to 670,000 by 2021. At the same time, the city has shifted from a strong industrial base to predominantly services and residential areas. This has resulted in changes in the structure of the urban development. The locations of former industrial plants are now occupied by modern housing estates, built among old residential buildings. This has contributed to an uneven and diversified heating structure with various sources of energy production. As a consequence, there are many problems with dust and gaseous pollution in the air. For this reason, the city of Łódź was chosen as an example to study the impact of changes in the context of a local, detailed analysis of air quality.

Air quality is a particular issue in urban environments^5–7^, where more than half of the world’s population lives (56%). According to the World Health Organization (WHO), air pollution in cities is the 13th major cause of death in the world^8^. The basic air quality parameters are particulate matter PM_10_ and PM_2.5_^9,10^ and gaseous pollutants SO_2_^11–13^. An European Environment Agency (EEA) report^14^ shows that the residential, commercial, and institutional sectors are responsible for 43.5% of PM_10_ emissions, with the manufacturing and extractive industry contributing an additional 22.4%, road transport 9.8%, and the energy industry only 5.4%. The residential, commercial, and institutional sector is responsible for 61.7% of PM_2.5_ emissions, with manufacturing and extractive industry contributing an additional 13.2%, road transport 10.5%, and the energy industry only 4.1%. According to the European Union (EU) Directive^15^, the permissible average daily concentration of PM_10_ is 50 µg/m^3^. According to the WHO^16^, the permissible limit for the average daily concentration of PM10 is 45 µg/m^3^. In the case of PM_2.5_, according to the EU the permissible annual average concentration level is 20 µg/m^3^ (no permissible daily level is specified). However, according to WHO the permissible average daily level of PM_2.5_ is 15 µg/m^3^. It should be remembered that particulate matter in urban areas is generated primarily by anthropogenic factors^17,18^. This is particularly important due to the fact that PM may contain harmful heavy metals produced by the combustion of fossil fuels^19,20^.

The combustion of fossil fuels is also associated with the emission of gaseous pollutants in urban spaces. Energy production is responsible for 64.8% of SO_2_ emissions, while the residential, commercial, and institutional sectors are responsible for an additional 11.7%, and the manufacturing and extractive industry is responsible for 20.8%^14^. This makes the measurement of SO_2_ concentration particularly important in urban areas, where there are high concentrations of possible pollutant emitters^21,22^. The permissible SO_2_ concentration according to EU guidelines^15^ should not exceed 350 µg/m^3^ (hourly average). As a gas heavier than air, SO_2_ often accumulates near ground level, which is particularly dangerous for human health^23,24^.

Hydrogen sulphide (H_2_S) is a highly poisonous gas released into the atmosphere through a range of human-related activities, notably from sewage systems^25,26^ as well as from industrial processes^13^. The detection threshold for H_2_S is only 0.14–1.42 mg/m^3^ (0.1–1 ppm). Even low concentrations of hydrogen sulfide < 10 mg/m^3^ in the air may cause eye irritation, while exposure to concentrations in the range of 70–140 mg/m^3^ (50–100 ppm) leads to neurological disorders^27,28^. The permissible level according to Polish national regulations^29^ is 0.02 mg/m^3^ (approximately 0.01 ppm, daily average). In the USA, the EPA specifies a permissible concentration of 0.001 ppm, with permissible concentrations of 0.75 ppm and 0.33 ppm for temporary exposure times of 10 min and 8 h, respectively^30^. The WHO has set the permissible level of H_2_S at 0.15 mg/m^3^ (approximately 0.10 ppm, daily average).

Currently, air quality monitoring in Europe is based on a system of stations measuring the concentrations of selected pollutants located mainly in cities (according to EEA data^31^). For example, in Berlin (Germany) there are 12 measuring stations (1 station per 74 km^2^); in Madrid there are 25 stations (1 station per 25 km^2^); in the city of Łódź (in central Poland) there are 3 measuring stations (1 station per 98 km^2^)^31^. Such large areas covered by single stations allows for only a general analysis of air quality. The detailed local analysis presented by us in this article is an innovative approach to the issue of air quality in agglomerations.

Detailed local analysis, especially in dense urban structures, is particularly important due to the complexity of the movement of pollutants and changes in their concentrations in the air. The direction and force of the wind have a particular impact on the dispersion of pollutants^32^. The uneven spatial locations of buildings of different sizes and shapes may cause zones of increased wind speed, turbulence, or air stagnation. This can contribute to the accumulation of pollutants^33^, or to the creation of so-called street canyons^34,35^. It should be emphasized that pollution in cities comes mainly from low emissions^36,37^—i.e., from emitters up to 40 m above the ground. Low emissions are responsible for urban smog and have a direct negative impact on the health and quality of life of residents.

In this study, we examine the air quality within a revitalized, post-industrial urban area in Łódź, Poland. The aim of the described analysis is to check the impact of technical changes (heat sources, thermal modernization) and revitalization of buildings in the agglomeration special economic zone on air quality. The complexity of the issue of air quality in highly urbanized areas makes local studies of air pollution concentrations a very important source of information^38,39^. Local studies use can make use mobile measuring instruments^40,41^, unmanned aerial vehicles (UAV)^42,43^, or small stationary devices^44,45^. Such research is particularly important for city residents, urban planners, urban space planners, and local authorities, as it can inform urban renewal strategies and renovation projects with the purpose of improving living conditions including air quality^46–48^. However, the research presented in the literature has a basic limitation resulting from the use of low-density sensors located only at one height from the ground, and therefore the inability to study the zones of pollution movement between/above buildings. It should be emphasized that the research presented in this article constitutes an innovative, comprehensive approach to the analysis of air quality in urbanized areas, assuming the use of UAV for measurements in 3D space at various heights along with computer spatial interpolation of the results and comparison of the results with an urban analysis of potential sources of pollution.

## Methodology

### Analyzed objects

**Figure 1:**
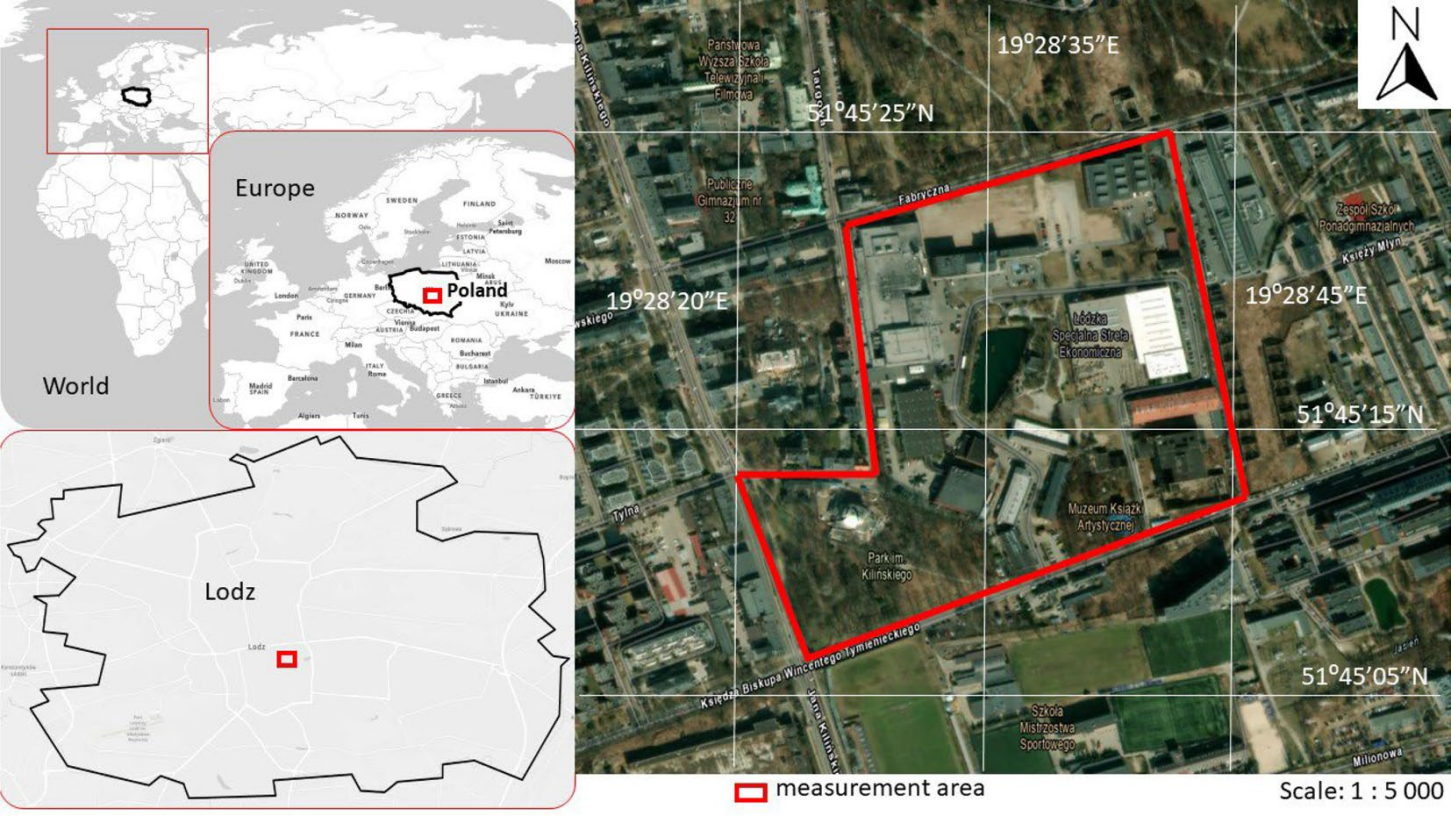
Location of the analysis area (background source^50^).

The subject of the analysis (Fig. 1) is an area belonging to the Lodz Special Economic Zone (LSEZ) covering 0.18 km^2^ of land (51°45′15" N, 19°28′35" E) in the center of the city of Łódź in central Poland (central-eastern Europe). The LSEZ provides preferential terms for businesses, including tax exemption (state financial aid)^49^. This area was chosen because it is an example of a revitalized post-industrial space, with modern infrastructure (access junctions, renovated buildings, and new constructions) which is intended to embody and represent the concept of sustainable development in urban areas. This includes the issue of improving air quality, which is the main focus of this study. In the last decade, Łódź has been undergoing significant economic and social changes. Once known predominantly for its cotton and clothing industry, and dominated by old-town buildings with individual heat sources, the city is changing into an area of investment in modern technologies and business zones. At the same time, the urban structures are being revitalized and modernized in line with sustainable development principles.

The industries in the analyzed LSEZ area currently include manufacturers of food, bakery products, plastic packaging, and high voltage disconnectors, as well as service desk providers, a fitness facility, restaurants, and a brewing plant. The LSEZ area also includes relaxation and rest zones, in the form of a park and a water reservoir.

**Figure 2:**
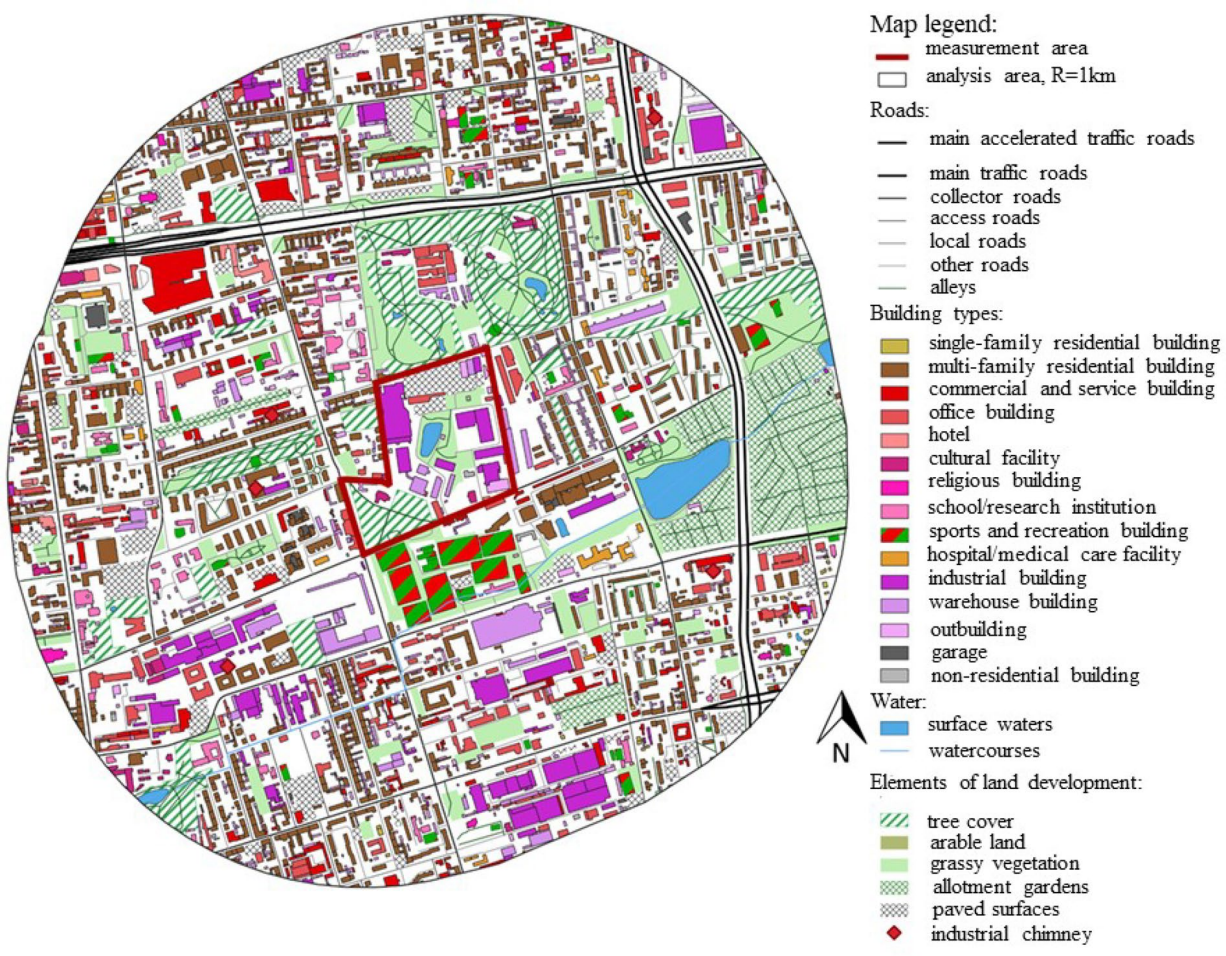
Map of the near vicinity (R = 1 km) of the LSEZ. Division of buildings according to their function. (Background source^51^).

Within a distance of 1 km from the analyzed area, buildings with a height of 4–8 storeys dominate. These include old town buildings for multi-family housing, post-industrial buildings and warehouses, and modern buildings for collective housing, as well as commercial and sports facilities (Fig. 2). In the immediate vicinity (R < 0.5 km) of the LSEZ, occupying post-industrial areas in the process of revitalization, there are multi-family residential buildings. These are mainly tenement houses along the streets and much newer housing estates between the streets, occupying post-industrial areas in the process of revitalization. The types of buildings that predominate in the LSEZ area are industrial and warehouse buildings, which are located mainly in the south. However, in the LSEZ area there are only buildings with industrial, warehouse, utility, and office functions.

**Figure 3:**
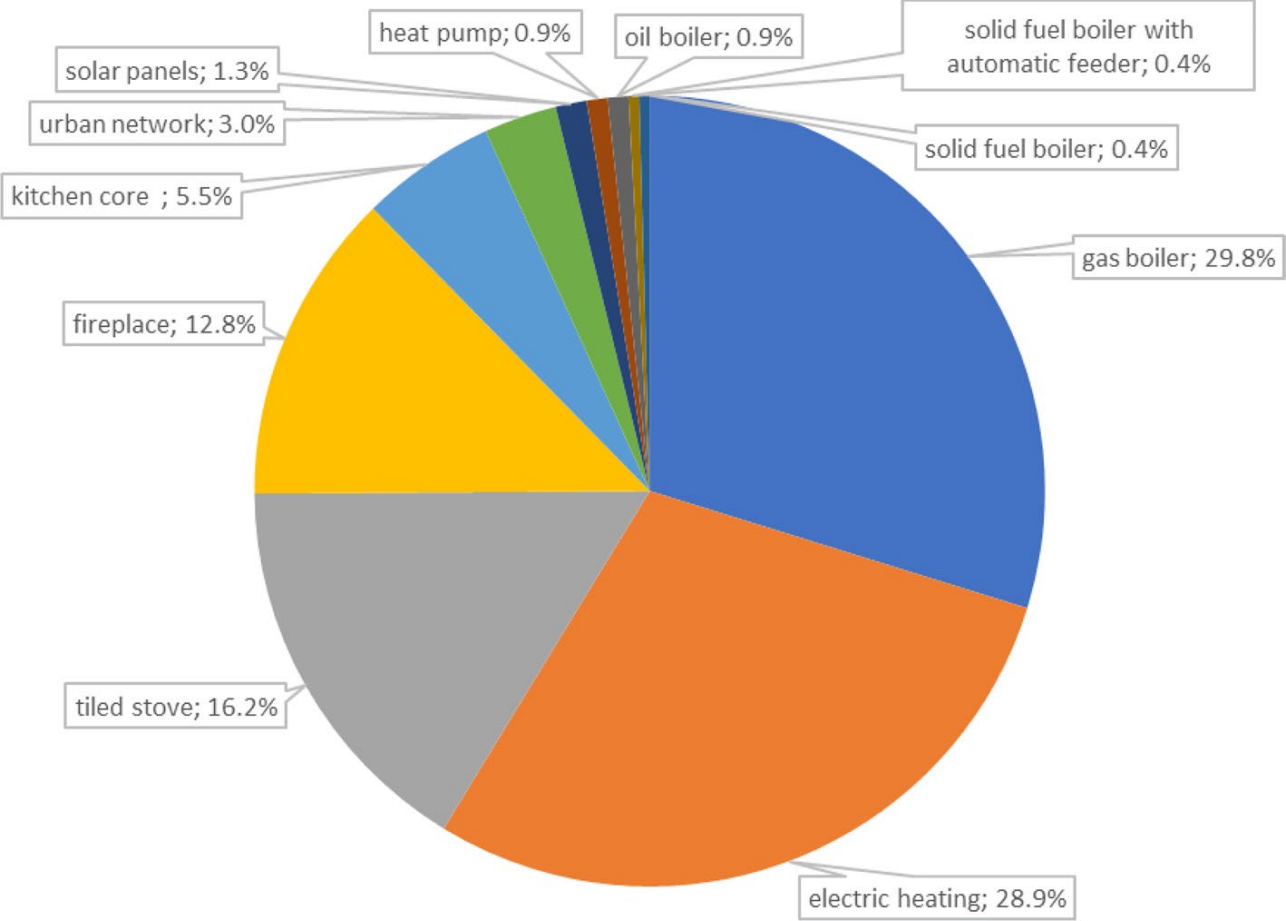
Types of heat sources in the near vicinity of the LSEZ area (own work, data source^53^).

Within 1 km around the analyzed LSEZ area, the dominant sources of heat energy are gas heating (29.8%) and electric heating (28.9%) (Fig. 3). This situation results from technical and economic modernization and changes in heat sources in old, multi-family tenement buildings. However, traditional heating sources based on solid fuels are still used, such as heating with a tiled stove (16.2%), fireplace (12.8%), or kitchen stove (5.5%)^52^. These ineffective heat sources represent significant local pollutant emitters in the city.

In the LSEZ area, the dominant sources of heat energy are natural gas (80%), the city heat pipeline (10%), and heat pumps or electric heating (10%)^53^. This is due to the revitalization of the area and the general policy of replacing solid fuels with ecological energy sources.

### Methodology of analysis

**Figure 4:**
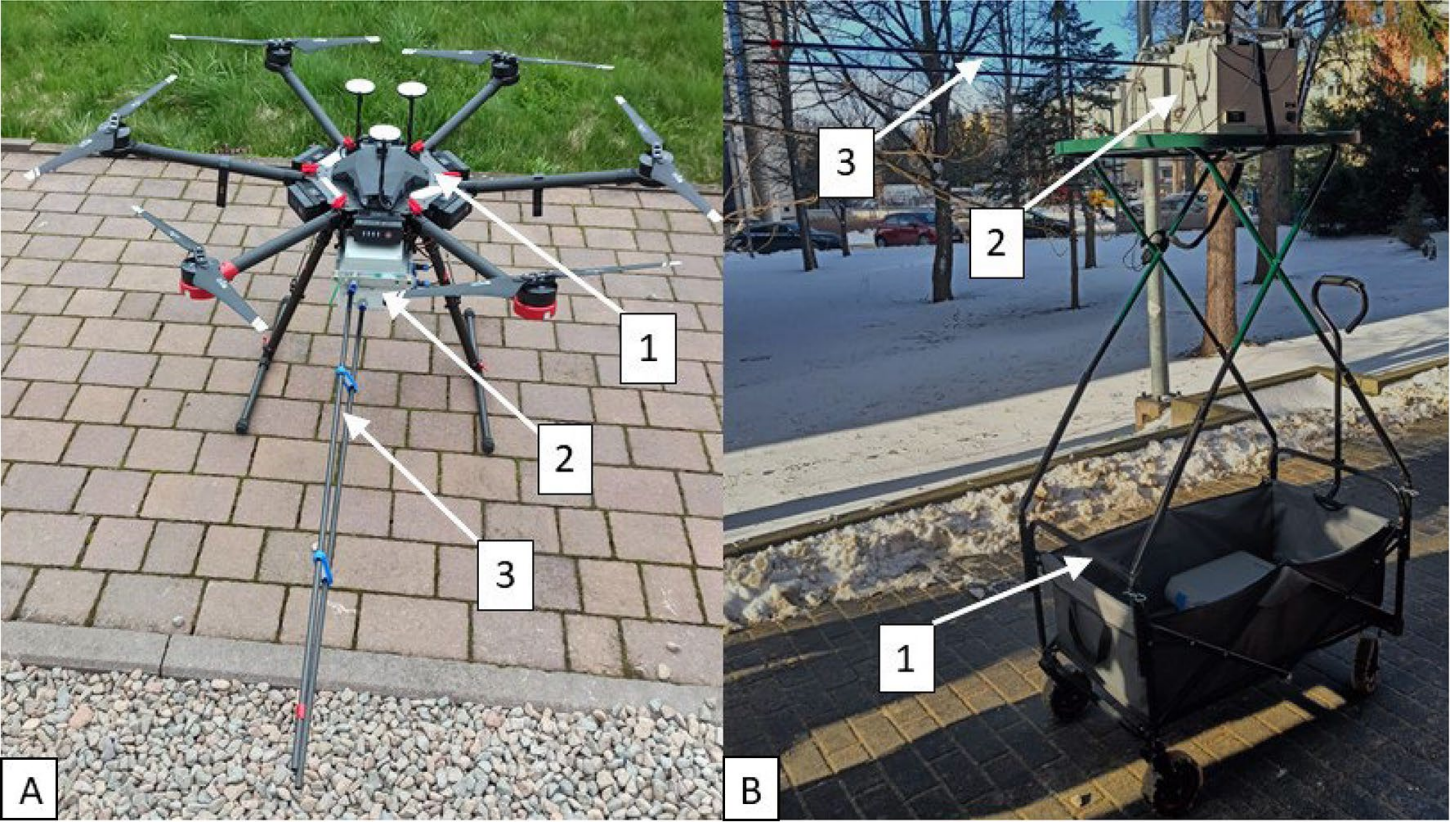
Measuring apparatus: 1A—unmanned aerial vehicle (UAV), 1B—transport platform (TP), 2—measuring equipment, 3—sampling probe.

The methodology was based on field measurements of pollutant concentrations in a selected area. The results were processed using 3D spatial interpolation in Arcgis Pro 3.2^50^. Field measurements were performed between January and February 2023, from 2 p.m. to 5 p.m., using measurement and sampling equipment installed on an unmanned aerial vehicle (UAV) (Fig. 4A) and a transport platform (TP) (Fig. 4B). The use of a UAV allowed for measurements to be made at heights ranging from 10 to 50 m above ground surface. The TP was used for measurements at a height of approximately 2 m above ground level.

The period between January and February 2023 was chosen because this is the “winter/heating period” in Central and Eastern Europe, when high exceedances of air quality standards are often recorded (up to 400%)^54^. Conducting measurements from 2 p.m. to 5 p.m. allowed us to take into account the impact of increased road transport and pollutant emissions from heating systems. Since the state of air quality is dependent on many factors, temporary, and highly variable, three representative/averaging measurement series were selected for further comparison. The selected series represent the most common scenarios of pollutant dispersion under the meteorological conditions in the period from January to February 2023. This allows for clearer visualization and comparison of 3D measurement results.

We measured PM_10_ and PM_2.5_, as well as SO_2_ and H_2_S gaseous pollutants. Particulate matter was selected because it is the primary pollutant analyzed worldwide^15,16^. The gaseous pollutant SO_2_ was selected due to the presence of numerous emission sources, in the form of heating systems. The concentration of H_2_S was selected due to reports from users of the LSEZ of unpleasant odors in the air.

The UAV with TP was equipped with a Laser Scattered (LS) sensor for measuring PM_10_ and PM_2.5_ (10,000 particles per second) and ElectroChemical (EC) type sensors for measuring H_2_S (3 ppb–1 ppm) and SO_2_ (0.5–2000 ppm).

The LS sensor for measuring PM was validated against data from provincial air quality measurement stations, which use measuring equipment based on reference methods (TEOM-FDMS). The ElectroChemical sensors were validated against measurements made using a VEGA-GC microchromatograph gas chromatograph equipped with a thermal conductivity detector (TCD), minimum concentration 500 ppb (0.005 ppm).

Numerical analyses of pollutant dispersion were performed using the ArcGis Pro 3.2^50^ program. Interpolation of the pollutant concentration distribution was carried out based on the Empirical Bayesian Kriging 3D method. The Kriging method was chosen over other interpolation methods, such as Inverse Distance Weighting (IDW)^55,56^ or Triangulated Irregular Network (TIN)^57,58^ methods, because it has the advantage of treating the observed variable as a random variable^59,60^. The weight coefficients are estimated by minimizing the sum of squared deviations for regression and using spatial autocorrelation (semivariogram). This improves the quality of spatial prediction^61^. The results were transformed into a 3D spatial image using the Voxel method to graphically display the actual measurement data^62^.

### Meteorological conditions

The months of January and February have the lowest average monthly air temperatures in Poland. According to statistics, these months also have the largest number of exceedances of permissible concentrations of air pollutants. The selected measurement series (Table 1) exemplify meteorological conditions typical for January and February 2023, and demonstrate the most interesting observed air quality states. Series I shows the prevailing wind direction (SW–W), wind speed (1–2 m/s) and air temperature (0.1 °C). Series II differs from Series I primarily in the direction of the wind, which affects the movement of pollutants within the LSEZ and adjacent areas. Series III differs from Series I in wind direction, and from Series II in wind speed (1 m/s higher).

**Table 1:** Summary of meteorological data (own work, data source:^63^).

Month/series	Temperature			Relative humidity			Wind speed			Dominant wind direction
	(2 m above gnd)			(2 m above gnd)			(10 m above gnd)			
	°C			%			m/s			°
	Min	Mean	Max	Min	Mean	Max	Min	Mean	Max	Mean
January	− 4.4	3.1	16.4	52	88	98	0	3	11	225
February	− 12	1.2	10.3	44	82	97	0	4	13	270
Series I	0	0.1	0.2	91	91	92	1	1.5	2	260
Series II	− 0.5	0.3	0.6	60	63	68	1	1.3	2	142
Series III	1.4	2.4	3.1	86	88	90	2	2.3	3	140

According to IMWM data^63^, the average area air temperature in January 2023 in Poland was 2.9 °C. This is 4.0 °C higher than the long-term average for the month (climatological normal period 1991–2020). January 2023 should be considered extremely warm for the time of year. The average area air temperature in February 2023 in Poland was 1.5 °C, which was 1.6 degrees warmer than the long-term average for the month (climatological normal period 1991–2020). February 2023 should be classified as a slightly warm month. These temperatures were probably influenced by climate change and global warming. Air quality research during warming is crucial to enable evaluation of the effects of activities aimed at improving air quality.

**Figure 5:**
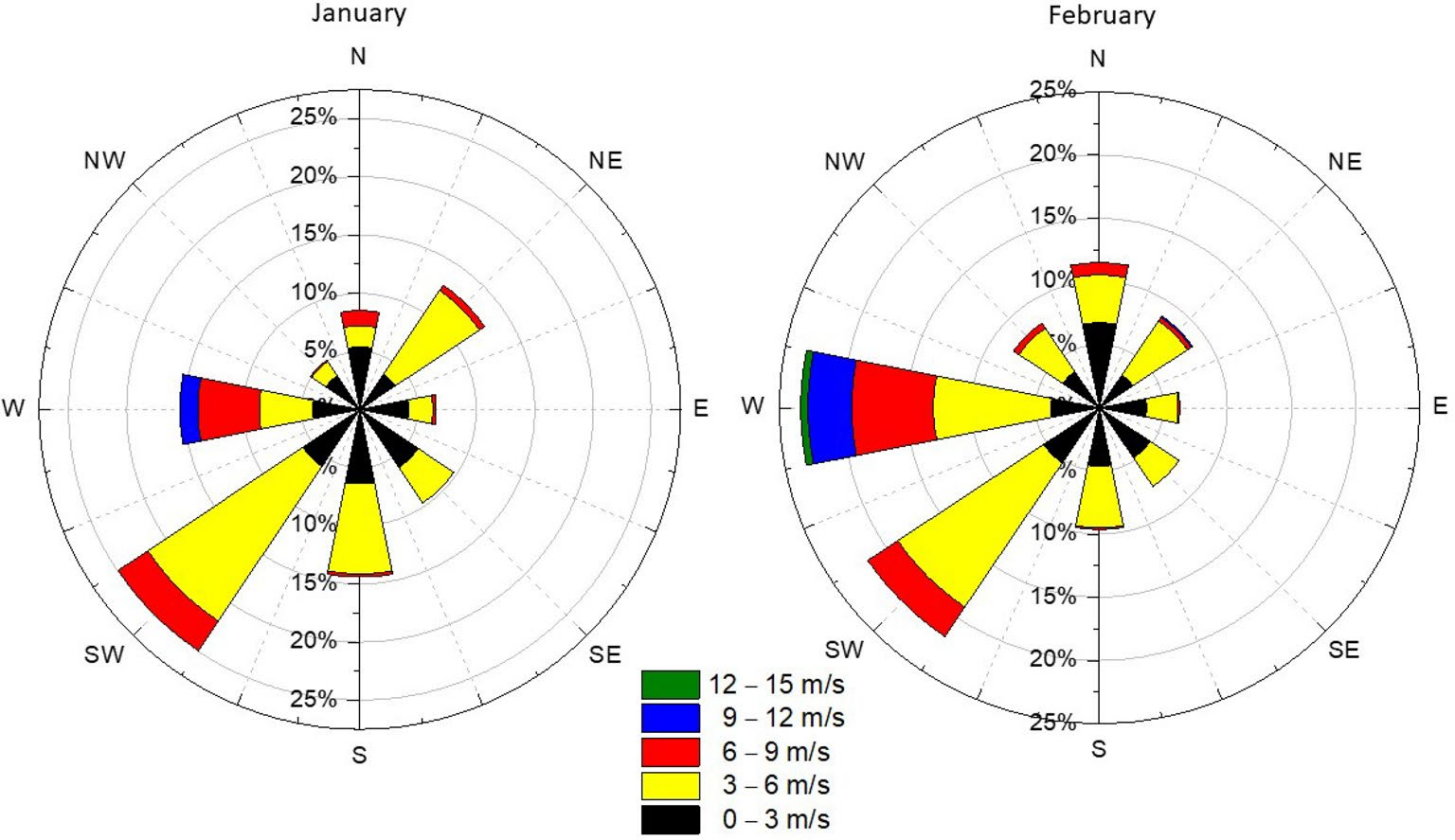
Wind Rose for January and February 2023 (own work, data source^63^).

In January 2023 in Łódź, the prevailing wind was from the SW (25%) (Fig. 5). In February, the prevailing wind was from the W (23%) (Fig. 5). The dominant wind directions (SW, W) are associated with the movement of air masses from the city center towards the analyzed area. This was taken into account in Series I. In contrast, for Series II and III we selected periods when the predominant wind direction was SE, which occurred very rarely (10% in January and 7.5% in February).

## Results

**Figure 6:**
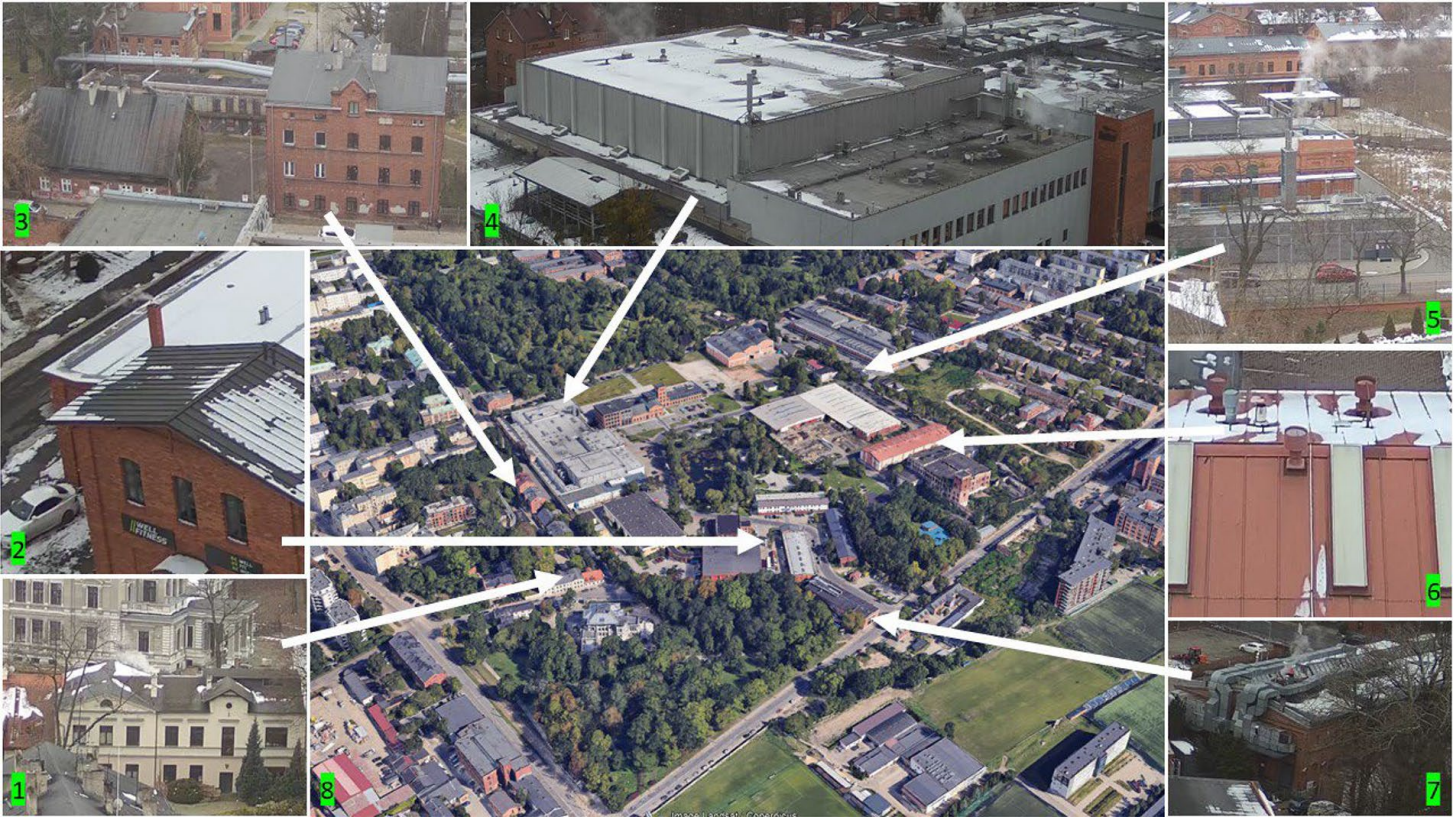
Potential sources of air pollution. 1–7: images from the UAV camera; 8: image from Google earth.

Figure 6 presents examples of sources of air pollution recorded during a UAV flight on the premises and in the immediate vicinity of the LSEZ. In the LSEZ area, the dominant sources of pollution are powered by city gas. Visible emitters adjacent to the LSEZ area are probably powered largely by solid fuels.

During the analysis period in January and February 2023, pollutant concentrations in the LSEZ area varied widely (Table 2). The highest concentrations of pollutants were recorded in February (Table 1), because February had a lower average temperature than January. Lower air temperatures in February contributed to higher demand for heat energy and, therefore, higher emissions. However, in terms of average concentrations, the analyzed area was characterized by worse air quality in January 2023. This may be related to the lower average monthly wind speed in January compared to February, which could translate into a stronger accumulation of pollutants in built-up areas. It should be emphasized that both minimum and maximum concentrations of pollutants refer to local zones and areas (they are point/local emissions). In Series I and II, we recorded a similar range of pollutant concentrations, despite the different wind directions. However, in Series III we recorded higher concentrations of pollutants, which were probably related to the stronger winds compared to Series I and II.

**Table 2:** Summary of measurement data.

Month/series	PM_10_			PM_2.5_			H_2_S			SO_2_		
	µg/m^3^			µg/m^3^			Ppm			Ppm		
	5-perc	Mean	95-perc	5-perc	Mean	95-perc	5-perc	Mean	95-perc	5-perc	Mean	95-perc
January	9.5	26.3	53.2	7.4	18.8	41.0	0.004	0.061	0.105	0.001	0.200	0.610
February	4.5	21.1	93.3	3.1	15.6	75.7	0.005	0.058	0.110	0.001	0.180	0.580
Series I	13.7	23.1	33.0	10.7	17.3	25.4	0.005	0.044	0.095	0.008	0.249	0.565
Series II	12.9	19.8	27.3	8.9	14.8	21.9	0.013	0.060	0.097	0.001	0.245	0.449
Series III	47.4	70.1	86.6	28.4	50.2	75.2	0.034	0.068	0.100	0.001	0.170	0.349

The spatial analysis of differences between individual series reveals interesting correlations between the structure of buildings, the locations of emitters, meteorological conditions, and air quality.

**Figure 7:**
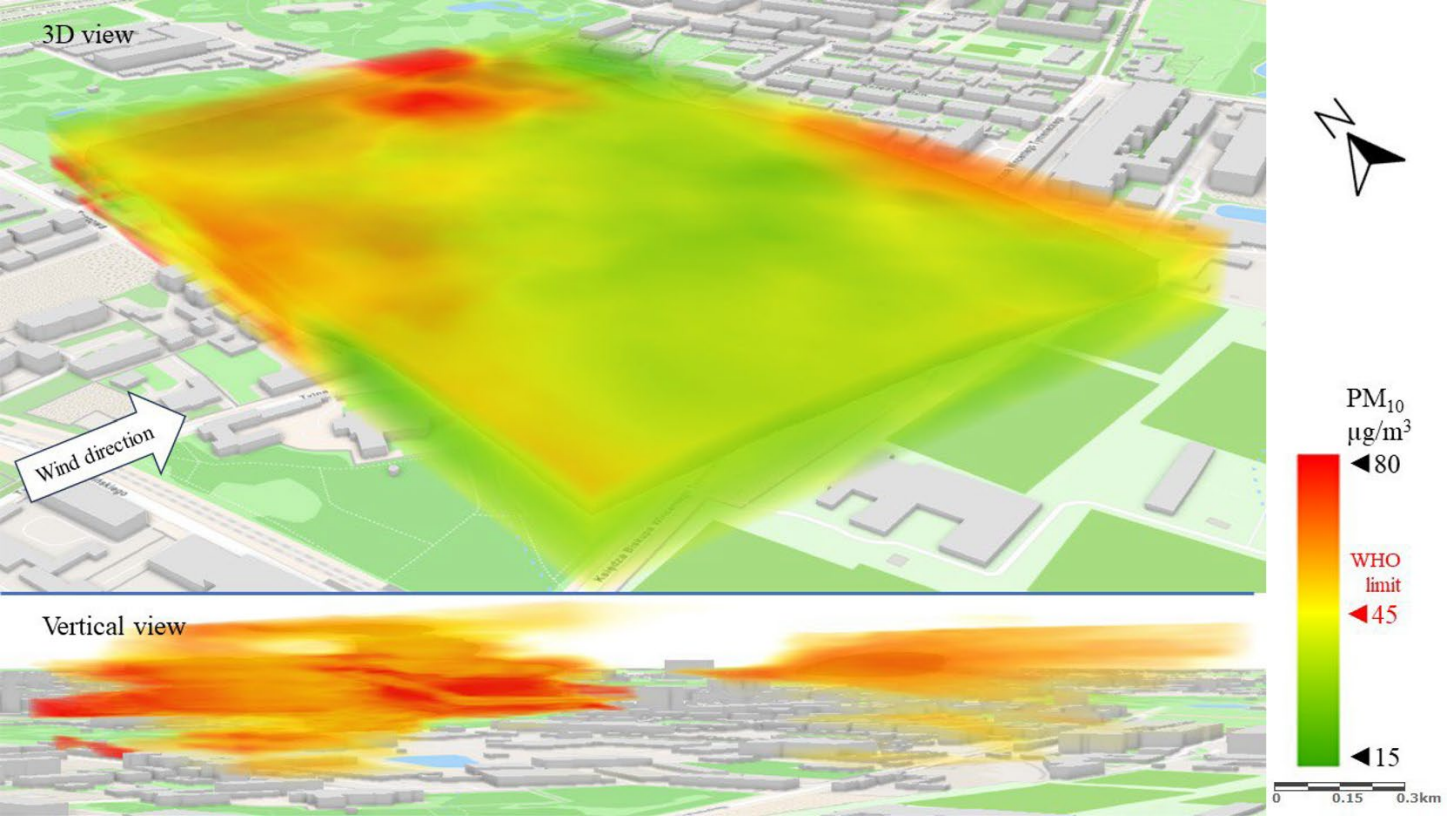
3D dispersion of PM_10_ pollution in Series I.

Series I represents a typical scenario for the spread of pollutants in the analyzed area, with the wind blowing from the city center (SW–W). The spatial image of PM_10_ pollution presented in Fig. 7 shows a strong division of the area into zones of higher and lower concentrations of pollutants. This observation was only made possible by the mobile and UAV technology used in the study. PM_10_ concentrations above the WHO limit^16^ of 45 µg/m^3^ were recorded in the north-western part of the LSEZ, in the area of old tenement buildings and a large industrial plant. A vertical 3D image shows that PM_10_ moved from the lowest parts of the troposphere upwards, where it spread with the wind to the north and over the north-eastern part of the analyzed area. Research by Sirithian and Thanatrakolsri^64^ found that both domestic and transboundary transport of pollutants from emitting areas could have contributed to the PM pollution problem in the urban and rural areas studied. Which proved that wind direction has a strong influence on the dispersion of PM pollutants. Low concentrations (Fig. 7) of PM_10_ (15–30 µg/m^3^) occurred in the southern part of the area, which to the west (from the wind direction in this scenario) is adjacent to a wooded area and modern housing estates.

**Figure 8:**
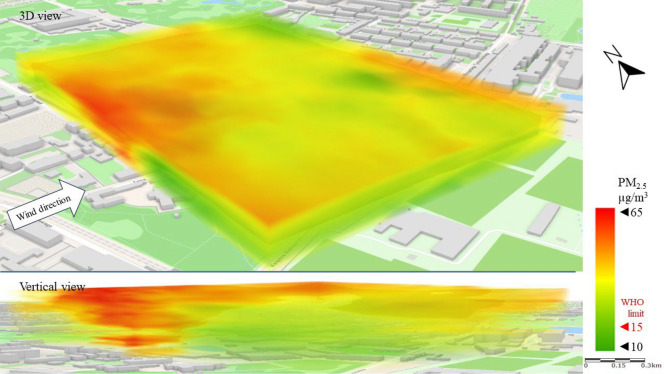
3D dispersion of PM_2.5_ pollution in Series I.

Figure 8 shows the emission and spread of PM_2.5_ (a lighter particulate matter fraction) in the direction of the wind. The emissions observed in the west of the LSEZ cause PM_2.5_ to rise in the atmosphere and then move over the LSEZ area in the upper analysis zones at an altitude of 40–50 m. It should be emphasized that in this repeated emission scenario, PM_2.5_ concentrations are four times higher than the WHO^16^ limits (15 µg/m^3^). This poses a much greater threat to human health and quality of life than the PM_10_ emissions. The open central space of the LSEZ and the lack of visible internal emitters provide effective ventilation of the area, diluting the concentration of pollutants and lifting them to higher parts of the atmosphere. At ground level, the concentration of PM_2.5_ in the LSEZ does not exceed the permissible limit of 15 µg/m^3^.

**Figure 9:**
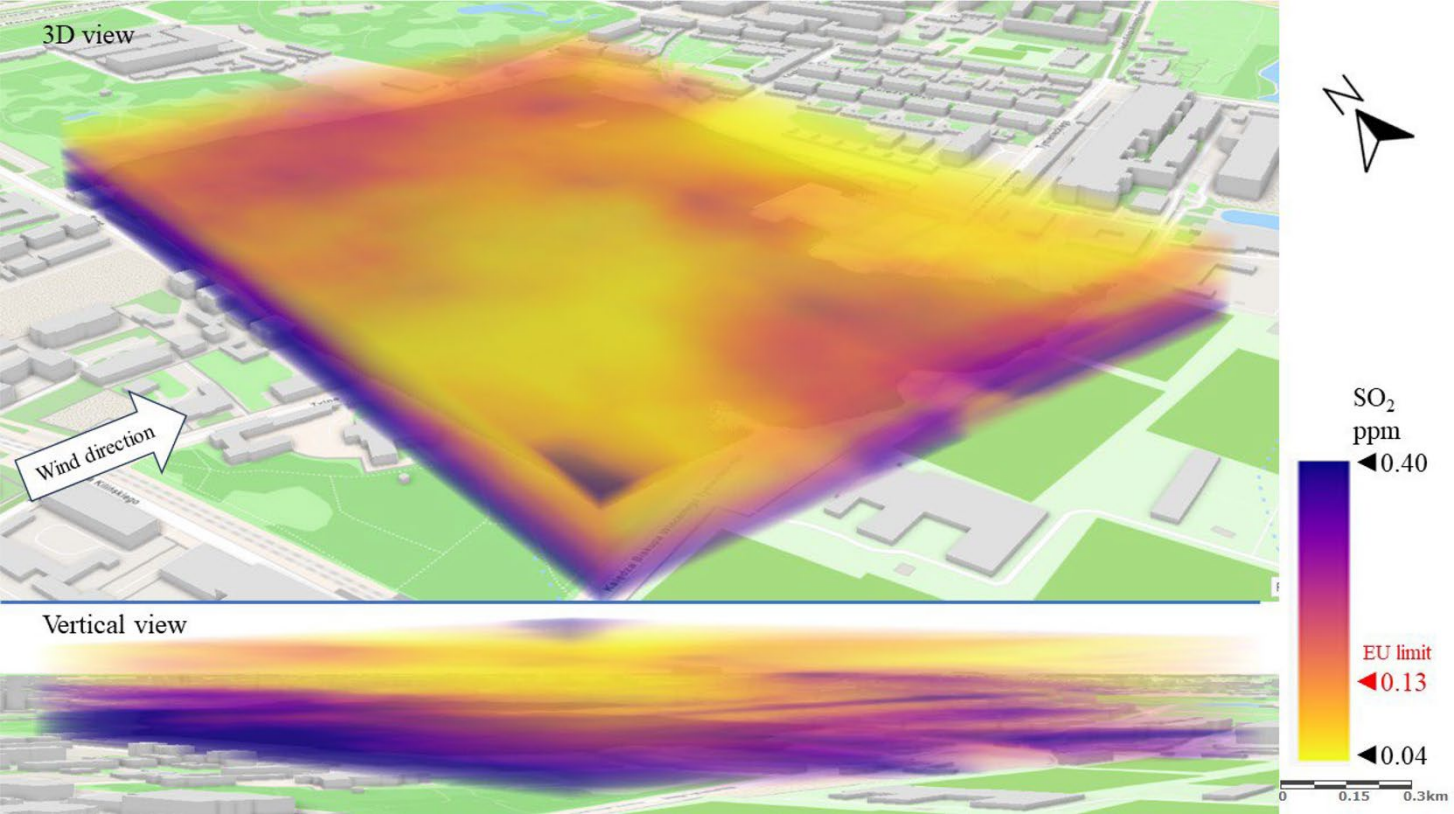
3D dispersion of SO_2_ pollution in Series I.

The next issue analyzed was gaseous pollution. Figure 9 shows the emission and dispersion of SO_2_ from the combustion of fuels for heat energy production. In Series I, reflecting the dominant wind directions over the city center, an accumulation of SO_2_ (a gas 2.5 times heavier than air) was observed at the ground surface to a height of approximately 15 m. In this zone, the SO_2_ concentration ranged from 0.15 to 0.40 ppm locally (western part of LSEZ), which was up to triple the EU limit^15^ of approximately 0.13 ppm. However, at more than 15 m above ground level the concentration was significantly lower, ranging from 0.04 to 0.10 ppm. This may result from the presence of low emitters—tenement houses at a short distance (especially on the western, windward side) and from the high roughness of the terrain, which resulted in local accumulation of pollution in the layer between the buildings. These observations are best illustrated by a vertical cross-section through spatial interpolation of pollution, which shows a rapid decrease in SO_2_ concentration in the area above the buildings compared to the ground level. Low wind speed (not exceeding 3 m/s) and high terrain roughness enhanced the accumulation effect.

**Figure 10:**
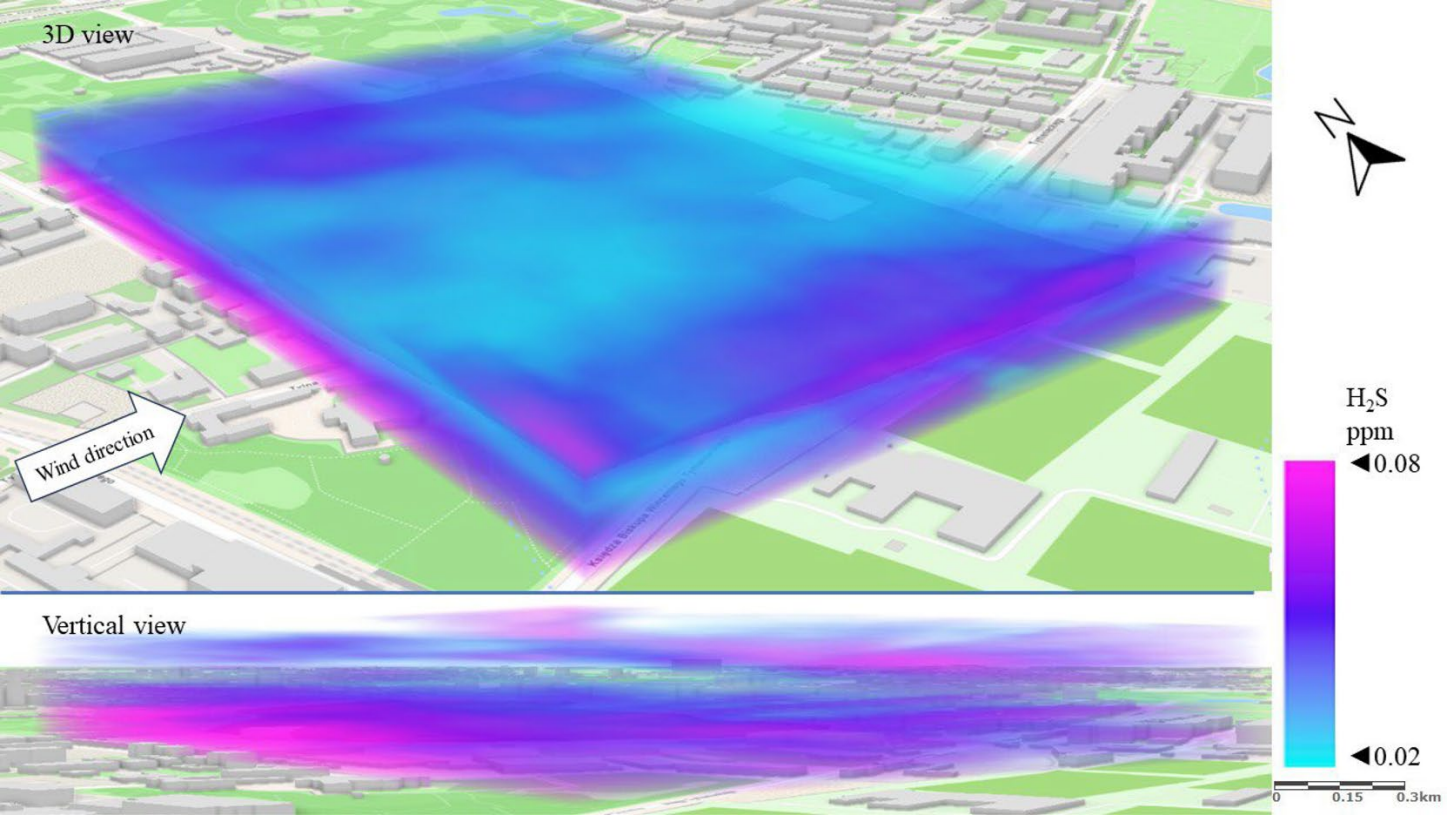
3D dispersion of H_2_S pollution in Series I.

Figure 10 shows a 3D dispersion map of H_2_S pollution in Series I. As a gas heavier than air, H_2_S accumulates near the earth’s surface. This phenomenon was observed in Series I. The highest H_2_S concentrations (above 0.05 ppm) were recorded in the streets around the LSEZ, especially on the west and north-western side. This is the area where the main sewage collectors are located and there are low-rise residential buildings, which are potential sources of H_2_S emissions. In the upper patios of the atmosphere (above 20 m), concentrations below 0.04 ppm were measured. The central area of the LSEZ, where the concentration was lowest, provides a visible canyon for ventilation of the zone, through an open space with a centrally located water pond.

The second graphically presented series represents periods of wind direction from the SE (opposite to the direction in Series I), with a speed comparable to Series I. It is therefore possible to observe how the dispersion of pollutants in the LSEZ changed with a completely different direction of pollutant transport—i.e., from open, green areas with loose development (within 0.5 km of the LSEZ).

**Figure 11:**
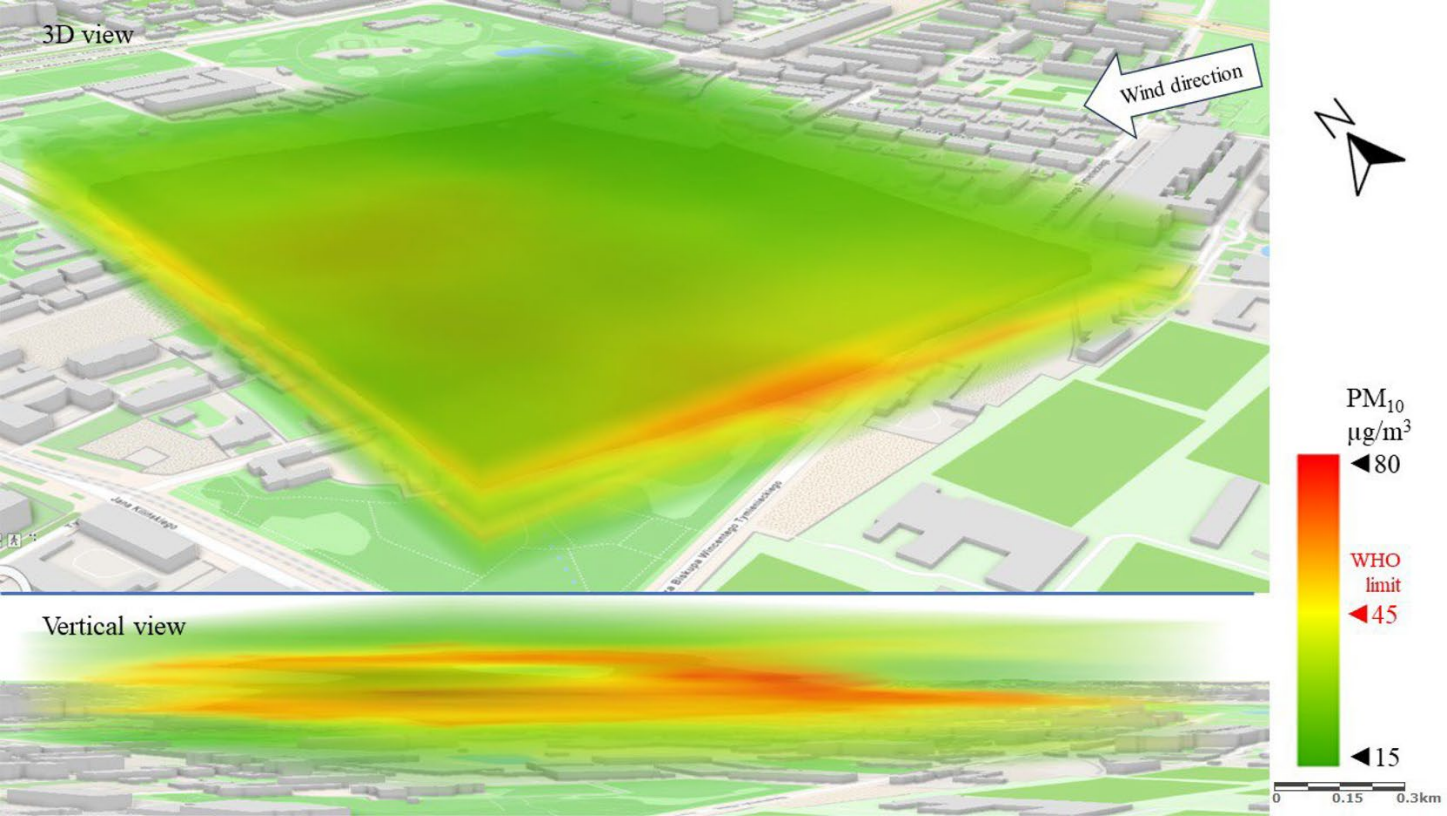
3D dispersion of PM_10_ pollution in Series II.

The different wind direction in Series II caused a significantly different spatial distribution of PM_10_ pollution than in Series I (Fig. 11). No clear emission area can be noted in Series II, only an inflow of pollution over the entire LSEZ area at an altitude of 10–30 m above ground level. The vertical cross-section of PM_10_ dispersion shows a concentration of 45–60 µg/m^3^ in narrow bands about 5 m high. The locally increased concentration of PM_10_ at given heights indicates the inflow of pollution with the wind from areas surrounding the LSEZ. It can be assumed that the PM_10_ emissions originate from a multi-family building with individual heat sources located 800 m away (in the direction of the wind). In the ground zone and at heights of 30–50 m, the concentration remained at 15–25 µg/m^3^.

**Figure 12:**
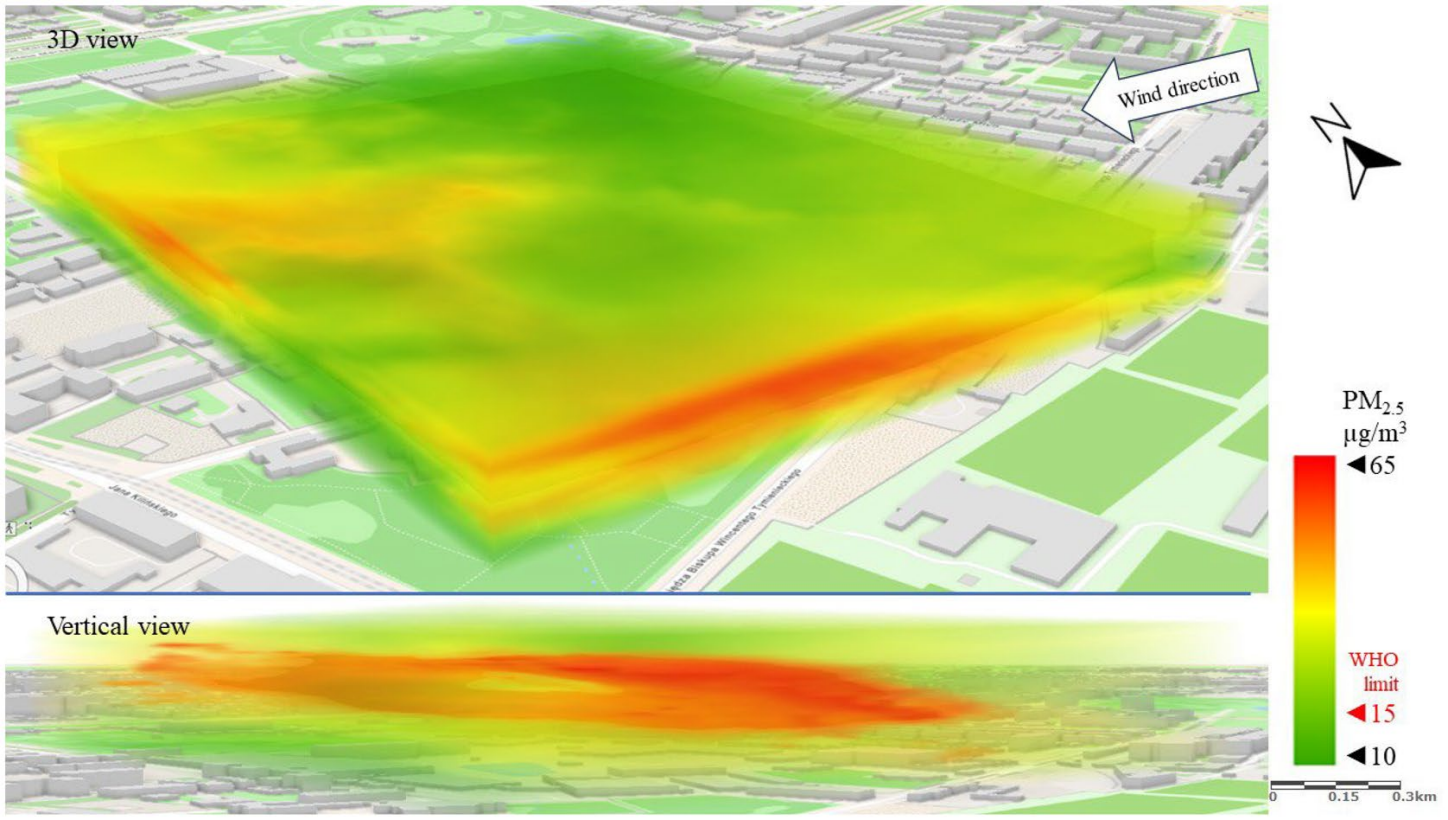
3D dispersion of PM_2.5_ pollution in Series II.

The movement of particulate matter pollution is clearly visible in the case of PM_2.5_ (especially in the vertical view). PM_2.5_ concentrations (Fig. 12) above the permissible limit of 15 µg/m^3^ moved from the SE of the area to the NW, gradually increasing in height. Similarly to Series I, a higher local exceedance of the permissible limit was recorded for PM_2.5_ than for PM_10_. It should be noted, however, that in most of the analyzed area the PM_2.5_ concentration was below 15 µg/m^3^.

**Figure 13:**
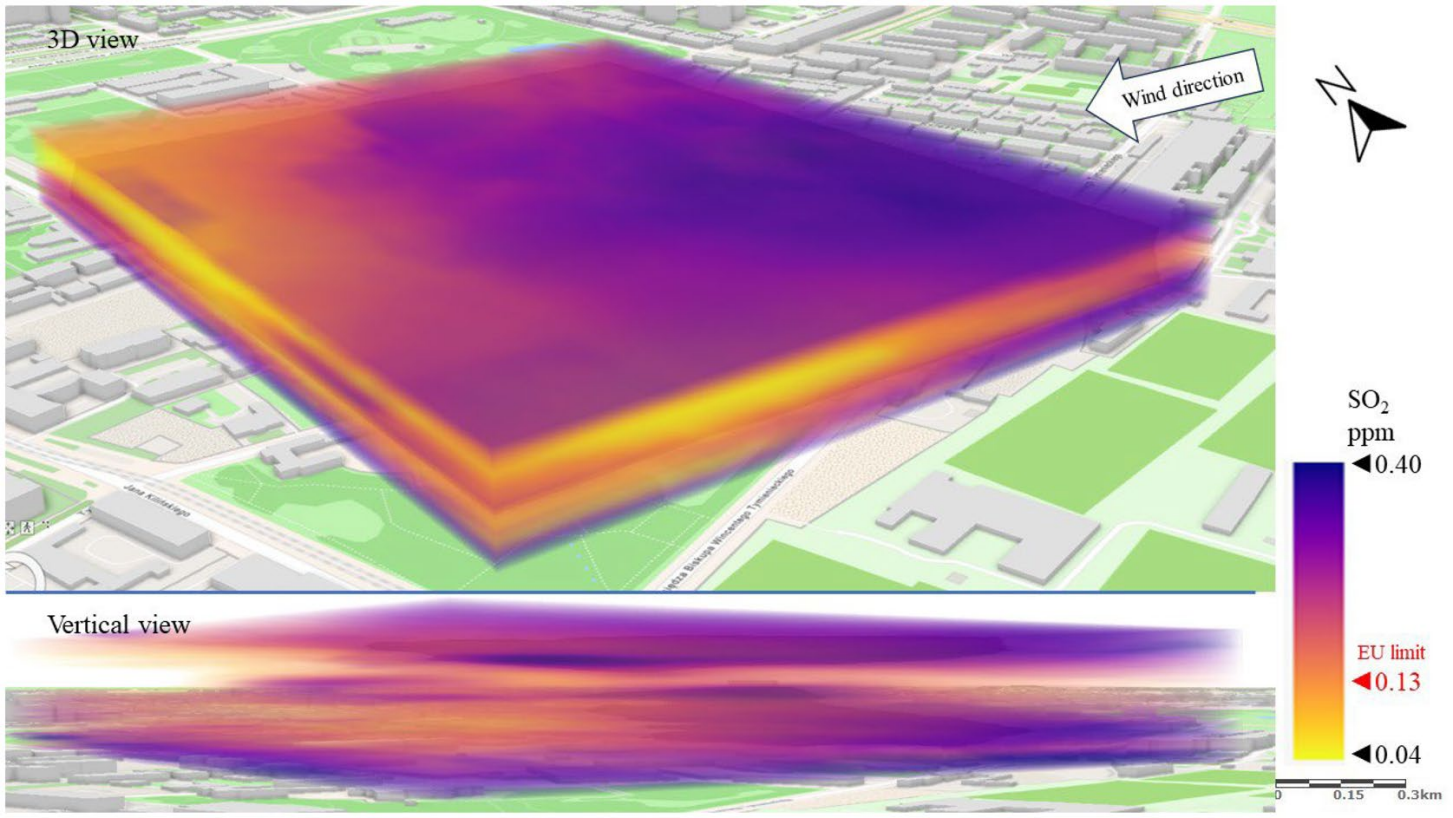
3D dispersion of SO_2_ pollution in Series II.

Figure 13 shows a 3D image of SO_2_ dispersion. The SE wind contributed to a three-layer division of the SO_2_ distribution. The highest SO_2_ concentrations (above 0.13 ppm) were recorded at an altitude of 40–50 m above ground level. This indicates that SO_2_ was transported with the wind from emitters distant from the analysis area. The direct source of the emission could not be established, but it was possible to observe the gradual dilution of the pollution from the SE to the NW of the LSEZ. The lowest SO_2_ concentrations (0.04–0.13 ppm) occurred at heights of between 20 and 40 m. This is an area of possible turbulence caused by the height of buildings (15–25 m). In the troposphere layer, between 5 and 20 m above ground level, another increase in SO_2_ concentration was recorded, probably related to the accumulation of falling pollutants.

**Figure 14:**
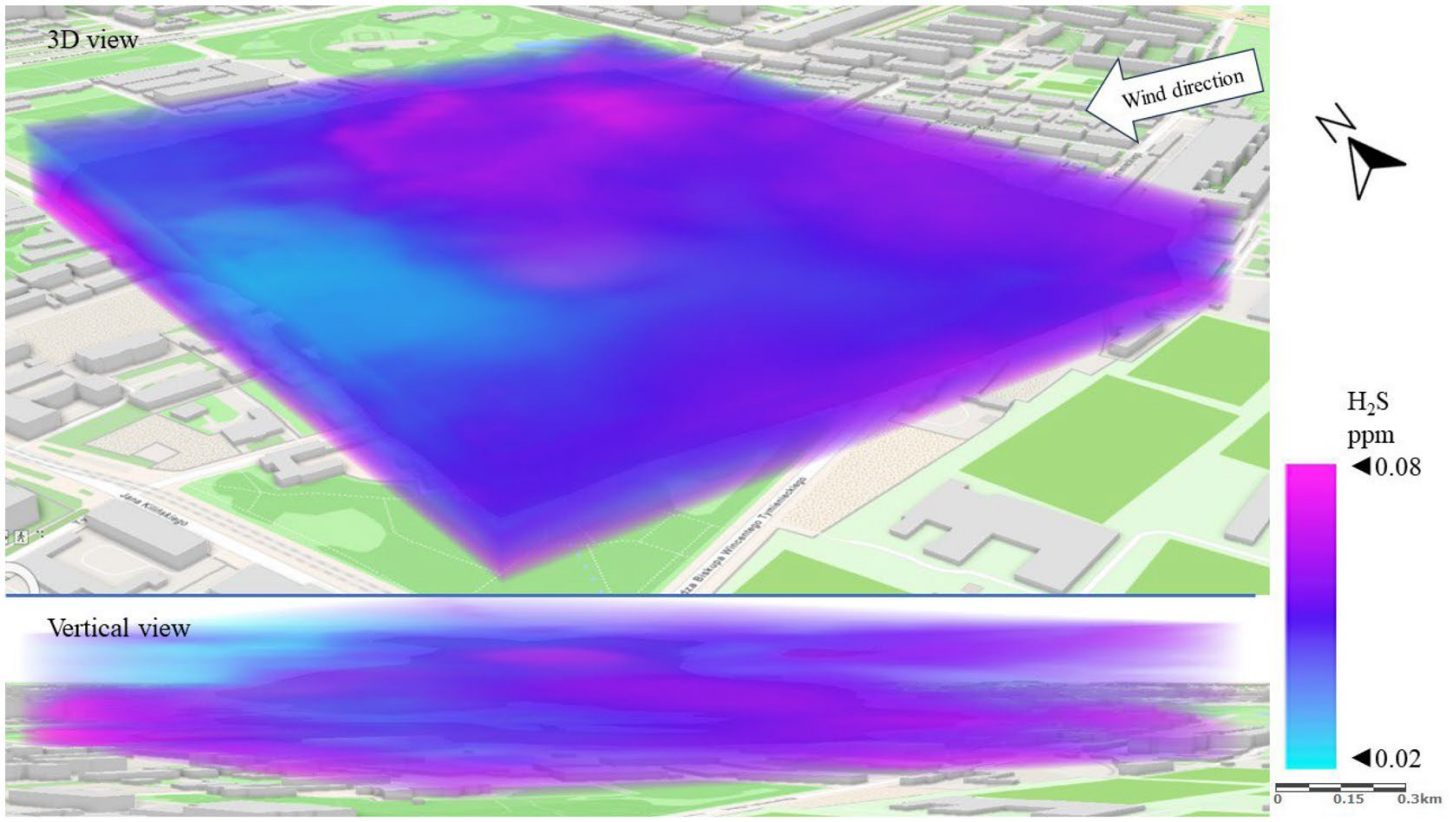
3D dispersion of H_2_S pollution in Series II.

Figure 14 shows the H_2_S dispersion in Series II. Again, increased concentrations of pollution are visible in the areas of roads and streets. The SE wind direction caused increased H_2_S concentrations in the western part of the LSEZ, which suggests that the source is the sewage system for the multi-family housing estate. A vertical view of the spread of pollution again revealed that H_2_S accumulated mainly at ground level between buildings, while open spaces contributed to lower its concentration. This is especially important because high concentrations of H_2_S can negatively affect human well-being.

**Figure 15:**
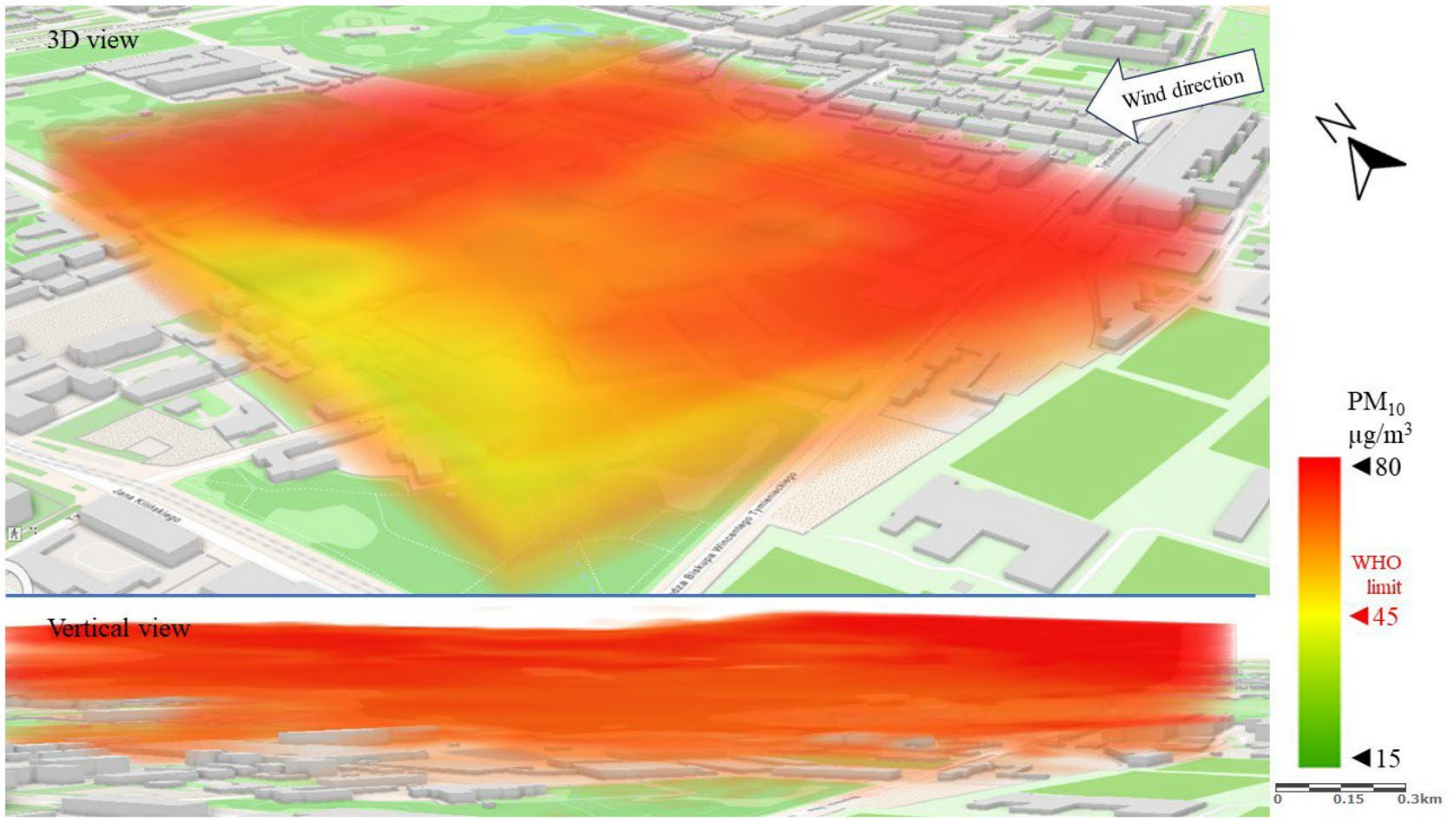
3D dispersion of PM_10_ pollution in Series III.

Series III demonstrates the significant impact of wind speed on the concentration and spatial dispersion of pollutants. As shown in Table 2, the highest concentrations of pollutants were recorded in Series III, when wind speed was almost twice as fast as in Series I and II. More importantly, these concentrations occurred in certain spatial layers of the troposphere. In the case of PM_10_, high wind speed contributed to concentrations from 60 to 80 µg/m^3^, mainly at heights above 30 m above ground level (Fig. 15). This proves the predominance of rapid transport of pollution from the surrounding areas to the LSEZ. The partially open, central space of the LSEZ contributed to local dilution of PM_10_ in the NW part, reducing the concentration to approximately 40 µg/m^3^.

In Series III, the PM_10_ concentrations in the ground level zone were in the range of 20–35 µg/m^3^, which is below the permissible level of 45 µg/m^3^ (Fig. 15).

**Figure 16:**
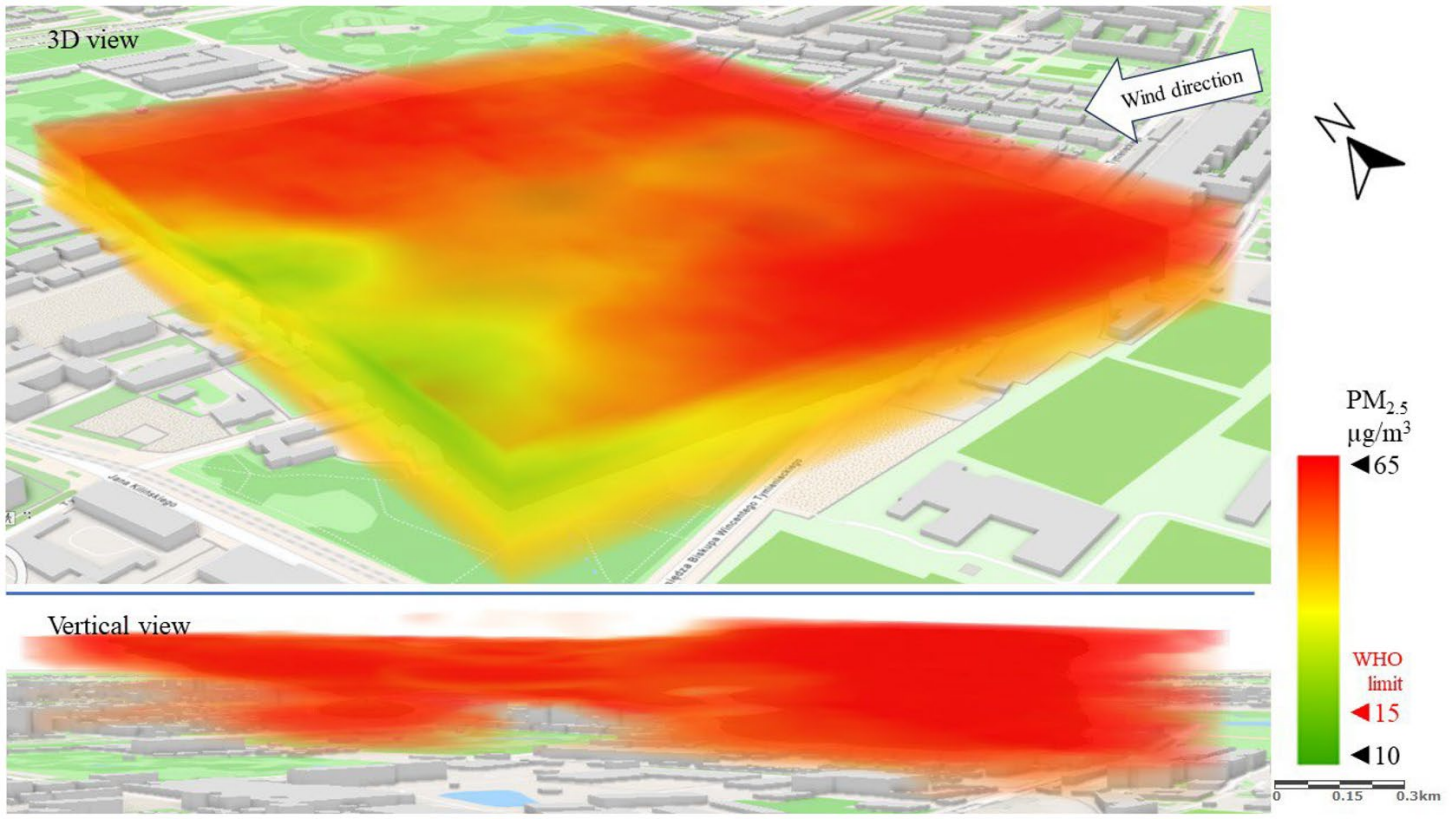
3D dispersion of PM_2.5_ pollution in Series III.

The impact of strong wind was also visible in the PM_2.5_ dispersion (Fig. 16). The highest PM_2.5_ concentration (> 35 µg/m^3^) was measured on the eastern (windward) side of the LSEZ. The pollution originated in open zones (streets, parking lots) at ground level and moved towards higher parts of the troposphere, moving over buildings at a height of over 30 m. High wind speed prevented it from falling and turbulence between buildings contributed to dilute the concentration of the PM_2.5_, as can be seen on the western (leeward) side of the LSEZ. Researchers Sekuła et al.^65^ analyzed the wind shear impact on PM_10_ vertical profiles in Kraków, and finding clear changes in PM_10_ concentrations at different heights from ground level. Which was caused by the disruption of air flow by the terrain and the variety of buildings.

Similarly to the concentration of PM_10_, the concentration of PM_2.5_ at ground level (vertical view in Fig. 16) did not exceed the permissible level (15 µg/m^3^).

**Figure 17:**
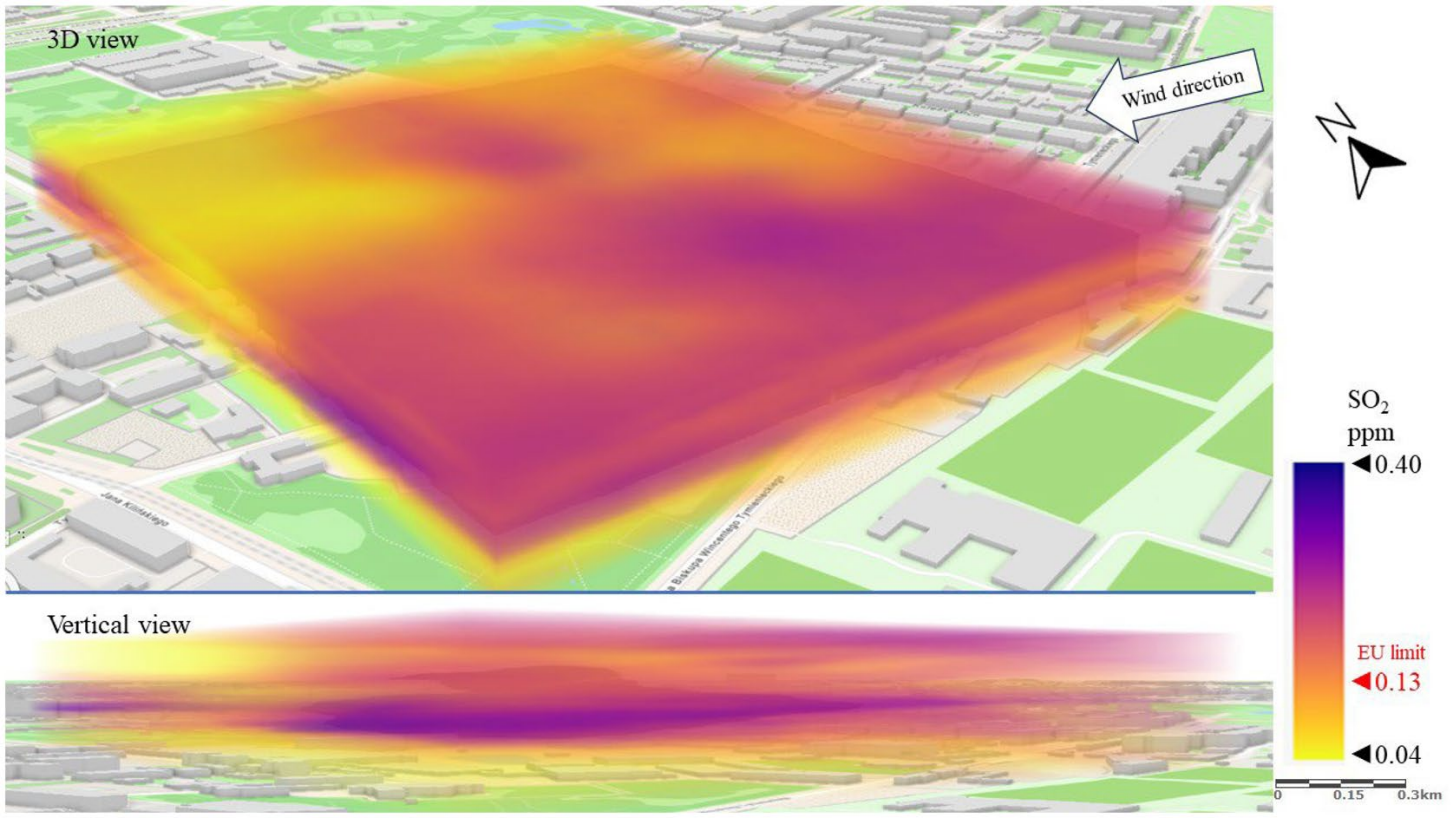
3D dispersion of SO_2_ pollution in Series III.

Based on our observations, wind direction and speed have a strong impact on the spread and concentration of gaseous pollutants. Similarly to Series II, the highest measured concentrations in Series III occurred mostly at altitudes above 30 m. This again indicates the transport of pollutants from areas adjacent to the LSEZ area. However, in the case of SO_2_ the almost twice higher wind speed in Series III (compared to Series II) resulted in a 30% reduction in the average SO_2_ concentration (Fig. 17). A strong negative correlation was found between wind speed and gaseous pollutant SO_2_ by Gupta et al.^21^. When the wind velocity doubled, the concentrations of pollutants decreased sharply to about a half. The dispersion of the pollutant visible in Fig. 17 shows a local reduction in SO_2_ concentration, especially at the higher analyzed heights. The lowest concentrations (< 0.13 ppm) were recorded in the north-western part of the LSEZ, where there is denser development. The turbulence between buildings probably helped to dilute the pollution. However, the highest SO_2_ concentrations were measured in the southern part of the LSEZ, where there are more wooded areas. This increases roughness, slows down air movement, and promotes the accumulation of pollution, even in the air above trees and buildings. Additionally, it was the windward side of the inflow of pollution over the LSEZ area.

The accumulation of pollution was clearly visible in the north-western part of the LSEZ, where the SO_2_ concentration reached a level of approximately 0.30 ppm, almost triple the permissible limit of 0.13 ppm. This area is adjacent to the park and covered with tall trees. Goyal and Sidhartha^22^ also proved that wind direction is related to SO_2_ concentration in Delhi. High SO_2_ concentrations were generally associated with the wind blowing from WNW–NW directions.

**Figure 18:**
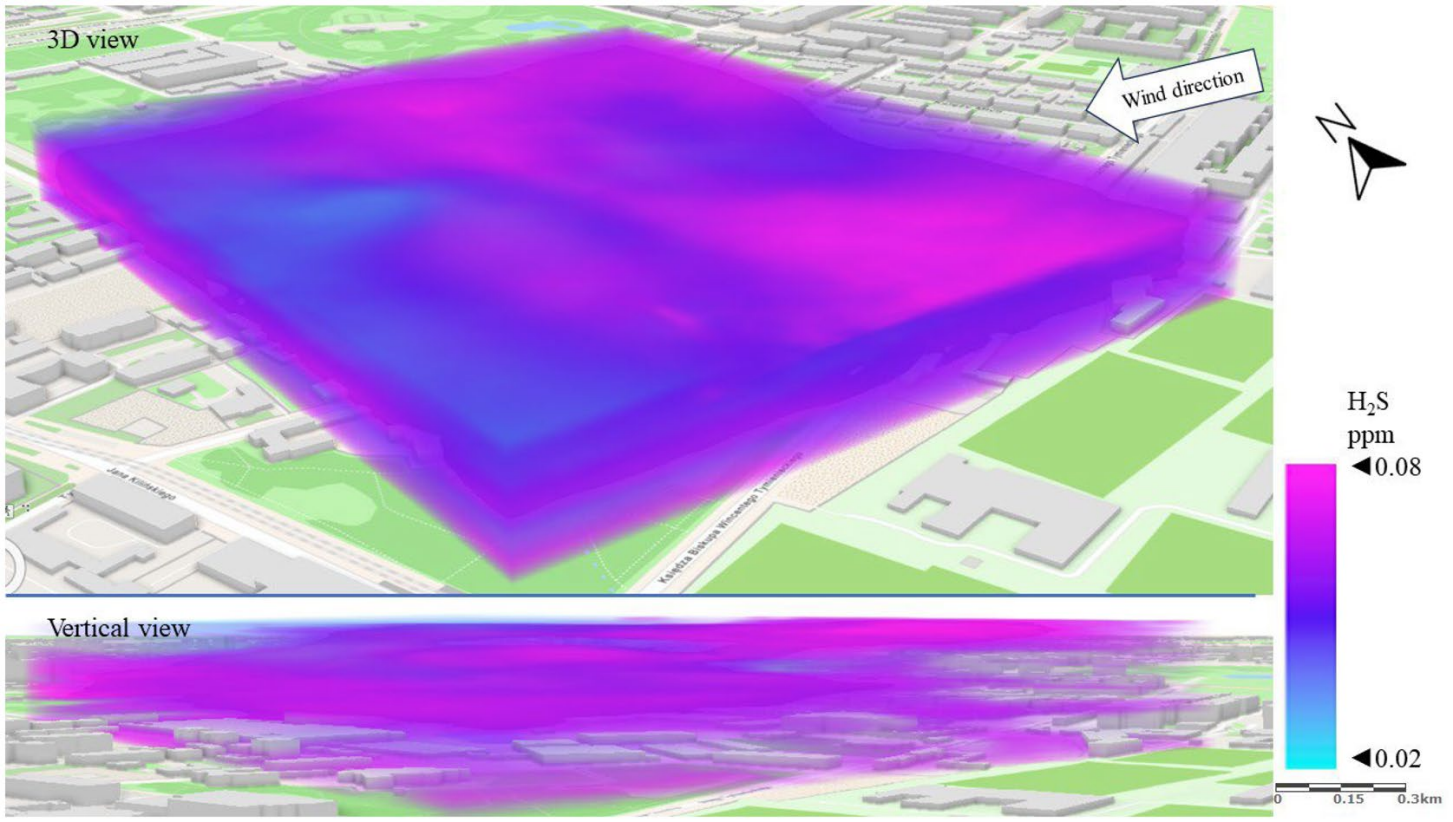
3D dispersion of H_2_S pollution in Series III.

The H_2_S dispersion in Series III resembles that in Series II. However, the stronger winds in Series III contributed to increase the average H_2_S concentration by 15%, especially at an altitude of 15–30 m above the terrain (Fig. 18). It is likely that the higher wind speed resulted in a larger pressure difference between the surroundings and the sewage stack, which produced greater gas emissions. This is related to the increased effect of chimney draft. The highest concentrations (> 0.06 ppm) of H_2_S occurred in the north-eastern and south-eastern sides of the LSEZ—i.e., from the side where the pollution was blown. However, the area of concentrations below 0.05 ppm decreased (compared to Series II), and was limited to the western part of the LSEZ at altitudes of 30 to 50 m.

## Conclusions

In this study, we have examined the air quality within a revitalized, post-industrial urban area in Łódź, Poland. The results show that the revitalization carried out in the LSEZ area contributed to eliminate internal air pollution emitters through the use of ecological and effective heat sources. However, emitters in the vicinity of the LSEZ caused air pollution, which was then transported over the area by the wind. This proves that wind direction and speed are key factors in the spread of pollutants. Therefore, it is impossible to really improve air quality in a given area without eliminating local emitters in the immediate vicinity. The research results indicate the need for annual monitoring of the analyzed area in order to confirm changes in air quality taking place in the city of Łódź through implemented ecological initiatives.

The use of UAV technology with mobile measurement equipment allowed for accurate assessment of air quality and factors influencing air quality on a local scale. The described methodology is universal and can be used anywhere in the world. It allows for the location of emitters, as well as for the identification of zones of accumulation or dispersion of air pollutants. The results can facilitate better urban planning and management of urban spaces.

In this study, no large-scale exceedances of permissible pollutant concentrations were observed in the analysis area. The exceedances were local, and concerned mainly the higher measurement zones of the troposphere (more than 30 m above ground level). In the case of PM_10_, the permissible level of 45 µg/m^3^ was exceeded twofold, and for PM_2.5_ the permissible limit of 15 µg/m3 was exceeded fourfold. However, these exceedances mainly concerned narrow areas of air pollution movement, at wind speeds above 2 m/s. Researchers Zhen et al.^66^ also observed that at wind speeds up to 2 m/s, the concentration of PM2.5 and PM10 was lower than at wind speeds of 4 m/s. Moreover, the wind direction had a clear influence on the PM concentration.

In the case of gaseous pollutants, higher wind speeds were associated with a decrease in the concentration of SO_2_ and an increase in H_2_S concentration. In both cases, the wind contributed to the occurrence of local areas of accumulation of these gaseous pollutants in the spaces between buildings or wooded areas. In the case of SO_2_, the permissible level of 0.13 ppm was exceeded locally by up to threefold. A 3D dispersion map showed that the location of increased H_2_S concentration coincided with the location of the sewage system and the sewage network of nearby buildings. However, the highest measured H_2_S concentrations did not exceed the detection threshold, which may indicate that the unpleasant odors reported by LSEZ users originate from another source. This will be the subject of further field research (Supplementary Information).

### Supplementary Information

Supplementary Information 1.

## Data Availability

Data is provided within the manuscript. Data files are available from the corresponding author upon reasonable request.
