# Supplementary figures and images for: Air quality in a revitalized special economic zone at the center of an urban monocentric agglomeration
Source: Sci Rep. 2024 Jul 5;14:15503. doi: 10.1038/s41598-024-66255-y (PMC11226688; doi:10.1038/s41598-024-66255-y)

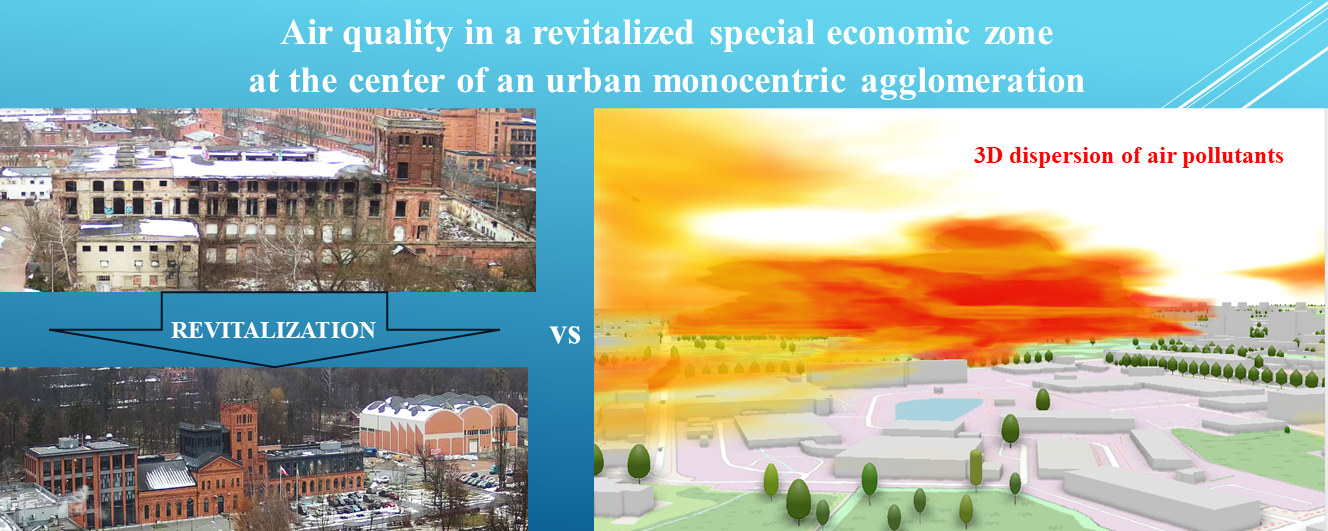

Supplement: Supplementary file 1 — Supplementary Information 1. [file 41598_2024_66255_MOESM1_ESM.png]
